# Interspecies Transmission of Reassortant Swine Influenza A Virus Containing Genes from Swine Influenza A(H1N1)pdm09 and A(H1N2) Viruses

**DOI:** 10.3201/eid2602.190486

**Published:** 2020-02

**Authors:** Helen E. Everett, Bethany Nash, Brandon Z. Londt, Michael D. Kelly, Vivien Coward, Alejandro Nunez, Pauline M. van Diemen, Ian H. Brown, Sharon M. Brookes

**Affiliations:** Animal and Plant Health Agency, Weybridge, UK

**Keywords:** interspecies transmission, influenza, influenza virus, viruses, influenza A(H1N1)pdm09 virus, swine influenza A(H1N2) virus, reassortant virus, pathogenesis, respiratory infections, zoonoses, pigs, ferrets, United Kingdom

## Abstract

Influenza A(H1N1)pdm09 (pH1N1) virus has become established in swine in the United Kingdom and currently co-circulates with previously enzootic swine influenza A virus (IAV) strains, including avian-like H1N1 and human-like H1N2 viruses. During 2010, a swine influenza A reassortant virus, H1N2r, which caused mild clinical disease in pigs in the United Kingdom, was isolated. This reassortant virus has a novel gene constellation, incorporating the internal gene cassette of pH1N1-origin viruses and hemagglutinin and neuraminidase genes of swine IAV H1N2 origin. We investigated the pathogenesis and infection dynamics of the H1N2r isolate in pigs (the natural host) and in ferrets, which represent a human model of infection. Clinical and virologic parameters were mild in both species and both intraspecies and interspecies transmission was observed when initiated from either infected pigs or infected ferrets. This novel reassortant virus has zoonotic and reverse zoonotic potential, but no apparent increased virulence or transmissibility, in comparison to pH1N1 viruses.

The ability of swine to support replication of phylogenetically diverse influenza A virus (IAV) strains from avian and mammalian origin poses a public health risk because of the potential for viral antigenic change resulting in variants with zoonotic properties (*1*). This risk was highlighted by emergence of the influenza A(H1N1)pdm09 (pH1N1) swine-origin influenza virus during 2009 (*2*).

In swine herds from Europe, the classical swine influenza virus A(H1N1) lineage 1A (*3*), was the only lineage detected before 1979. This strain has a common origin with the progenitor virus that caused the human 1918 influenza pandemic. However, in the early 1980s, the classical swine H1N1 strain was displaced by a new European enzootic swine IAV strain, the Eurasian, avian-like H1N1 (H1_av_N1) lineage 1C (*3*), probably after cross-species transmission directly from birds to mammals (*1*). H1_av_N1 virus underwent rapid and sustained adaptation to mammals, as shown by phylogenetic as well as phenotypic changes, including enhanced mammalian replication and transmission (*4*). This H1_av_N1 lineage is now enzootic and has also undergone reassortment resulting in emergence of multiple genotypes and 2 main enzootic subtypes, H1N2 (H1_hu_N2), also designated lineage IB (*3*), and H3N2, through acquisition of human seasonal influenza virus–origin hemagglutinin (HA) or neuraminidase (NA) gene segments (*1*).

Since the global dissemination of pH1N1 virus, this lineage has also undergone reassortment with swine IAV strains endemic to the corresponding geographic region (*5*). Within Europe, this diversification of pH1N1 virus in pigs has increased the circulating swine IAVs from 4 to >25 genotypes (*6*,*7*). Diversification has been rapid; pH1N1 reassortant viruses incorporating enzootic swine virus HA and NA, or NA alone, detected during 2010 in the United Kingdom (*8*) and Italy (*9*), have emerged. The NA segment was N2 subtype in both instances. Subsequently, viruses have been identified that contain the NA segment from European enzootic H3N2 strains and the remaining 7 segments from pH1N1-origin viruses (*10*,*11*) or a further reassortant containing H3 of human seasonal origin (*12*).

Reassortment between enzootic and pH1N1 influenza viruses was also rapidly detected elsewhere, including Asia, where a reassortant swine IAV containing pH1N1-origin NA was described during 2010 (*13*), and in North America, where several reassortants containing HA and NA segments from enzootic viruses have been maintained since 2011 (*14*). These swine IAV reassortants from North America incorporate different combinations of the pH1N1 internal gene cassette (gene segments other than HA and NA) and invariably carry the pH1N1-origin matrix (M) protein gene. 

Swine IAV H1N2 virus reassortment strains circulating in Brazil acquired NA gene segments from independent introductions of human seasonal H3N2 strains similar to those circulating in the late 1990s and an HA segment derived from human H1 strains circulating in the early 2000s. These lineages have subsequently reassorted with co-circulating pH1N1 strains (*15*) and viruses with a pH1N1-origin internal gene cassette have been isolated from wild boar (*16*) and swine (*17*). One such H1H2 reassortant gave rise to a human clinical case of influenza in a worker on a swine farm (*18*). A reassortant H1N2 virus strain was also isolated from pigs in Chile; this virus incorporated human-origin HA and NA gene segments prevalent in the 1990s and the pH1N1 internal gene cassette (*19*). Human infection with swine-origin H1N2 viruses (H1N2v infection) has been reported in the United States, most notable being 3 human influenza cases linked to infection with a related co-circulating H1N2 swine IAV virus incorporating a pH1N1-origin M gene segment (*20*).

Genetic reassortment between enzootic swine and pH1N1 strains giving rise to H1N2 virus diversification has also been reported in Asia, including lineages in South Korea containing Eurasian avian-like swine HA and Korean swine H1N2 NA gene segments and the pH1N1-like internal gene cassette (*21*) and another reassortant strain associated with pig respiratory disease in China incorporating the HA, basic polymerase protein 2, and M genes of swine pH1N1 virus origin; the remaining gene segments were derived from a swine H3N2 strain (*22*).

We characterized a prototypical reassortant swine influenza A virus, A/swine/England/1382/2010 (H1N2r) (*23*), which has become enzootic in the pig population in the United Kingdom (*6*). This virus incorporates the genes encoding the envelope glycoproteins, HA and NA, from a European swine H1N2 subtype (which themselves derive from human-origin strains) and the remaining gene cassette encoding the internal proteins from swine-origin pandemic 2009 strains (*8*). Serologic assessment of potential exposure of pig industry workers in the United Kingdom to swine viruses during 2009–2010 showed that antibodies to H1N2 swine IAVs were present in 24% of persons, and this prevalence was increased relative to a comparator population (*24*). Because the potential risks associated with a novel combination of gene segments in the H1N2r isolate were unknown, we investigated the pathogenesis and infection dynamics of this virus in pigs, the natural host, and in ferrets, which are widely established as an animal model for investigating influenza and pandemic risk in humans (*25*,*26*).

## Materials and Methods

### Viruses

We isolated H1N2r virus A/swine/England/1382/2010 from nasal swab specimens from pigs that had clinical signs of mild influenza-like disease (*8*). Virus was propagated in embryonated specific pathogen-free chicken eggs according to standard methods (*27*), passaged once to obtain a virus stock, and characterized by using whole-genome sequencing (*8*) using reference influenza A virus strains (Table).

**Table:** Influenza virus strains of swine or human origin used for analysis of swine influenza A virus containing genes from pH1N1 and swine influenza A(H1N2) viruses*

Virus strain	Abbreviation	Gene segment origin†		Use	Reference
HA + NA	Internal cassette
A/swine/England/1382/2010	H1N2r	H1_hu_N2‡	pH1N1	In vivo, in vitro	(*8*)
A/swine/England/1353/2009	Sw/pdm09	pH1N1	pH1N1	In vitro	(*28*)
A/swine/England/997/2008	H1_hu_N2‡	H1_hu_N2‡	H1_av_N1	In vitro	(*6*)
A/England/195/2009	Hu/pdm09	pH1N1	pH1N1	HI	(*29*)
A/swine/England/201635/1992	H3N2	H3N2‡	H1_av_N1	HI	(*30*)
A/swine/England/195852/1992	H1_av_N1	H1_av_N1	H1_av_N1	HI	(*31*)
A/swine/England/104270/2011	H1_hu_N2‡	H1_hu_N2‡	H1_av_N1	HI	(Animal and Plant Health Agency, unpub. data)

*Av, avian; HA, hemagglutinin; HI, hemagglutination inhibition; hu, human; NA, neuraminidase; pH1N1, influenza A(H1N1)pdm09; Sw, swine. †Gene segment origin refers to the virus lineage origin of gene segments encoding the HA and NA envelope proteins or the remaining gene segments of the internal gene cassette. ‡Swine virus gene segments previously derived from human-origin viruses.

### Organ Culture

Ferret organ cultures were prepared as described (Appendix). Organ cultures were inoculated by adsorbing virus for 1 h at 37°C onto the air interface of triplicate tissue sections. The culture was then washed and replenished with supplemented Dulbecco modified Eagle medium and incubated before virus detection 24–48 hours postinoculation (hpi) in combined supernatant and tissue lysate per sample. Control organ cultures were mock inoculated. Influenza A virus nucleoprotein (NP) was detected in tissues by using immunohistochemical (IHC) analysis as described (*32*).

### Real-Time Reverse Transcription Quantitative PCR Analysis

Virus RNA was extracted by using a QIAmp Viral RNA Biorobot Kit (QIAGEN, https://www.qiagen.com) and assayed by using a real-time quantitative reverse transcription PCR (qRT-PCR) (*33*). Virus RNA was quantified as relative equivalent units (REUs) (*34*) against a 10-fold dilution series of RNA prepared from the inoculum stock with a titer of 10^6^ 50% egg infectious dose (EID_50_)/mL. REU measures the amount of virus RNA present and not infectivity. However, it can be inferred from the linear relationship with the dilution series that REU values are proportional to the amount of infectious virus present.

We used Prism software (GraphPad, https://www.graphpad.com) for statistical analyses. We determined the area under the curve of the shedding profile for each animal and compared between groups by using the Tuckey multiple comparisons test and 1-way analysis of variance. Positive-sense RNA encoding the virus M gene was quantified by using a positive strand-specific real-time qRT-PCR (Appendix).

### In Vivo Studies

All animal studies were reviewed and approved by the Animal and Plant Health Agency Ethical Review Panel. Studies were conducted according to the Animal (Scientific Procedures) Act of 1986 and Animal Research: Reporting of In Vivo Experiments guidelines.

High health status, 6–8-week-old, male Landrace hybrid pigs and male fitch ferrets (weight range 750–1,000 g, maximum age 3 months) were bred in the United Kingdom. Before infection, all animals were confirmed free of influenza A virus RNA by performing real-time qRT-PCR analysis of nasal swab specimens (pigs) or nasal washes (ferrets) (*33*). These animals also showed negative results in a hemagglutination inhibition (HI) test (*35*) using 4 influenza A virus antigens (pH1N1, H1_av_N1, H3N2, and H1N2 viruses) appropriate for the pig population in the United Kingdom (Table). Animals were subcutaneously implanted with a biothermal idENTICHIP (Destron Fearing, http://destronfearing.com) for daily temperature monitoring, and a clinical scoring system was used for daily clinical monitoring (*36**,**37*).

### Experimental Infection

We intranasally infected animals by using protocols appropriate for the species and nasal structure. We infected pigs by using a MAD Nasal Intranasal Mucosal Atomization Device (Wolfe-Tory Medical Inc., https://www.lmaco.com) to deliver droplets with a diameter of 30–100 μm. A total dose of 10^6^ EID_50_ units diluted in phosphate-buffered saline to a final volume of 4 mL (2 mL/nostril) was administered. We anesthetized ferrets and infected them by intranasal droplet instillation of a total dose of 10^6^ EID_50_ in 0.4 mL inoculum (0.2 mL/nostril).

### Pathologic Analysis

For each species, postmortem examination was conducted for 4 animals at 3 days postinoculation (dpi) and 5 dpi, as well as for 2 animals at 7 dpi. Gross pathology was assessed and tissue samples were collected in 10% [vol/vol] phosphate-buffered formalin for histopathologic analysis. Lesions were scored as described for ferrets (*37*) and pigs (*36*). Influenza A virus NP was detected in tissues by using IHC analysis as described (*36**,**37*).

### Experimental Infection and Transmission Studies

Six animals of each species were directly infected for study I (intraspecies transmission) and study II (interspecies transmission). In study I, immunologically naive animals (n = 6) of the same species were placed in direct contact at 1 dpi. In study II, animals of 1 species (n = 6) were infected and immediately thereafter cohoused animals of the other species (n = 6) were reintroduced. Pigs were housed on the floor and immediately adjacent to ferret cages (minimum distance 30 cm), and the floor of the ferret cage was at the height of the heads of the pigs. Virus shedding was monitored daily from 1 dpi through 12 dpi in nasal swab specimens from pigs (1/nostril) or nasal wash samples from ferrets (1 mL/animal). Serum samples were obtained before infection and at the end of the study at 21 dpi. An overdose of barbiturates was used for euthanasia of all animals.

### Swab and Tissue Specimen Processing

Daily nasal swab specimens, which were pooled for each pig, were eluted in 1 mL Leibovitz L-15 medium with l-glutamine supplemented with 100 U/mL penicillin, 1,000 μg/mL streptomycin, and 1% fetal bovine serum (all from GIBCO). Ferret nasal wash samples were obtained by using 1 mL phosphate-buffered saline (GIBCO). Tissues were analyzed by preparing a 10%–20% [wt/vol] homogenate in 1 mL supplemented Leibovitz-15 medium by using an Omni GLH Homogenizer (Omni International, https://www.omni-inc.com).

### Serologic Analysis

Serum samples were analyzed by using an influenza A virus NP competitive ELISA (IDVet) according to the manufacturer’s specifications and dilutions of 1:40 for pig serum samples and 1:10 for ferret serum samples. Antibody titers to the H1N2r virus were measured by HI as described for pig (*35*) or ferret (*38*) serum samples.

## Results

### H1N2r Virus Ex Vivo Replication

**Figure 1:**
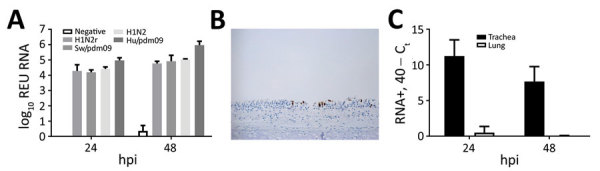
Infection and replication of swine H1N2r virus in ferret tracheal organ cultures. A) Quantity of influenza A virus RNA in ferret tracheal ex vivo organ cultures at 24 h and 48 h postinoculation with swine viruses H1N2r (A/swine/England/1382/2010), Sw/pdm09 (A/swine/England/1353/2009), H1N2 (A/swine/England/997/2009), and Hu/pdm09 (A/England/195/2009). Results are log_10_ REU in combined supernatants and tissue lysates for each sample. Error bars indicate upper end of SEM for triplicate cultures. B) Immunohistochemical labeling of influenza A virus nucleoprotein in ferret tracheal cultures infected with H1N2r reassortant virus (original magnification ×20). C) Detection of virus-positive strand replicative intermediate RNA in ferret tracheal and lung cultures at 24 and 48 h postinoculation with H1N2r virus. Error bars indicate upper end of SEM for triplicate cultures. C_t_, cycle threshold; hpi, hours postinoculation; hu, human; H1N2r, reassortant swine influenza A virus; pdm09, influenza A(H1N1)pdm09; REU, relative equivalent unit; sw, swine; +, positive.

To investigate H1N2r virus replication in the ferret animal model, we inoculated ferret organ cultures with virus strains at the same viral multiplicity of infection or mock-inoculated. We quantified influenza A virus replication by using real-time qRT-PCR for virus RNA at 24 hpi and 48 hpi. Replication of the H1N2r isolate was comparable to that of swine pH1N1 and H1N2 strains and a human-origin pH1N1 strain in tracheal cultures (Figure 1, panel A). We also detected comparable amounts of virus RNA in lung cultures at 24 hpi; these amounts decreased slightly by 48 hpi for some strains (Appendix Figure). We confirmed H1N2r virus replication in tracheal cultures by positive IHC labeling for influenza A virus NP (Figure 1, panel B). A strand-specific PCR detected replicating virus-positive sense RNA in infected tracheal cultures (Figure 1, panel C), which decreased between 24 hpi and 48 hpi, most likely reflecting virus replication kinetics. These in vitro assay results showed productive replication of the swine-origin H1N2r virus in ferret cells, but we observed no selective growth advantage linked to the novel gene constellation.

### Pathologic Assessment

We then investigated the pathogenicity profile after H1N2r infection of pigs, the natural host, or ferrets, which represent a surrogate model for human infection. We assessed pathologic changes at 3, 5, and 7 dpi in both species and observed gross pathologic changes at 3 dpi in 2 of 4 directly infected pigs. In 1 of these pigs, a large portion of a single lung lobe was consolidated by lesions consistent with influenza A virus infection. We also found gross pathologic changes in the lungs in all pigs at 5 dpi, with a reduction by 7 dpi. In infected ferrets, small sporadic lesions common to influenza A virus infection occurred, and individual animals had lesions in the respiratory turbinates (5 dpi) and salivary glands (7 dpi), as well as in the left cranial lung lobe in 2 directly infected ferrets at 3 dpi and 5 dpi.

**Figure 2:**
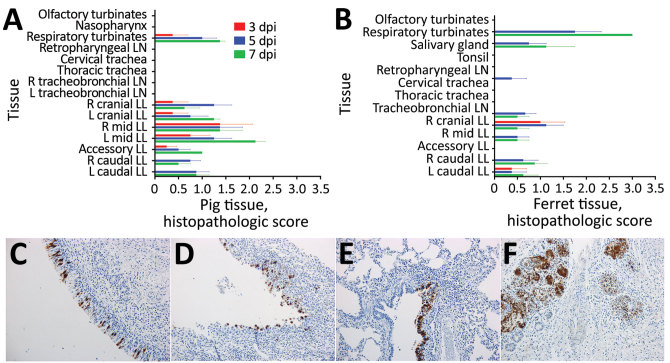
Histopathologic and immunohistochemical analyses of tissues from pigs or ferrets directly infected with swine influenza A(H1N2) reassortant virus showing mild disease. A, B) Histopathologic scores for pigs (A) or ferrets (B) are calculated as mean for 4 animals at 3 dpi and 5 dpi or 2 animals at 7 dpi. Error bars indicate SEM. Tissues are indicated in anatomic order from the upper to lower respiratory tract. C–G) Immunohistochemical labeling for influenza A virus nucleoprotein (brown) is shown for tissue sections from pig respiratory turbinates at 5 dpi (C), ferret respiratory turbinates at 7 dpi (D), pig lung at 5 dpi (E), and ferret lung at 7 dpi (F) (original magnification ×20 for C, D, E, and F). Ferret salivary gland tissue at 76 dpi (G) shows intense labeling (original magnification ×10). dpi, days postinoculation; L, left; LL, lung lobe; LN, lymph node; mid, middle; R, right.

Histopathologic changes were consistent with mild inflammatory disease. For pigs (Figure 2, panel A), inflammation was evident throughout the respiratory tract at all timepoints (e.g., in respiratory turbinates [upper respiratory tract] and lung lobes [lower respiratory tract]) at 3, 5, and 7 dpi. In contrast, for ferrets, most pronounced inflammation occurred in the respiratory turbinates and in the salivary glands, initially at 5 dpi and increasing by 7 dpi, which indicated a predominantly upper respiratory tract infection (Figure 2, panel B).

Influenza A virus NP labeling showed replicating virus in pig and ferret tissues, notably in pig respiratory turbinates, at 5 dpi (Figure 2, panel C). For pig lung tissues, we detected virus in the right mid lung at 3 dpi and in all lung lobes at 5 dpi (Figure 2, panel E), with evidence of virus clearing by 7 dpi. For ferret tissues, virus NP labeling was more pronounced at 7 dpi, as shown for respiratory turbinates (Figure 2, panel D) and lungs (Figure 2, panel F). We observed intense IHC virus labeling in salivary glands of ferrets. These animals showed an increase in virus labeling from 3 dpi through 7 dpi (Figure 2, panel G).

### H1N2r Virus Infection

**Figure 3:**
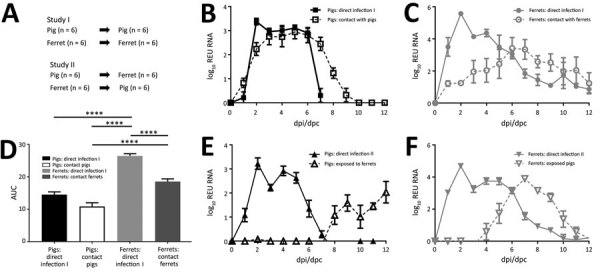
Nasal shedding and transmission of swine influenza A(H1N2) reassortant virus from infected animals. A) Schematic outline of study design. B, C) In study I, virus RNA in nasal swab specimens from directly infected or contact pigs (B) and directly infected or contact ferrets (C) was quantified by using real-time reverse transcription quantitative PCR. Values are indicated as REU at each dpi or dpc. Error bars indicate SEM. D) AUC for virus shedding profiles in study I in pigs or ferrets, showing significant differences (****p<0.0001) in virus shedding by directly infected ferrets compared with all other groups and between contact pigs and ferrets. Error bars indicate upper end of SEM. E, F) In study II, virus RNA in nasal swab specimens from infected or cohoused pigs (E) or nasal washes from infected or cohoused ferrets (F) was quantified as REU. Error bars indicate SEM. AUC, area under the curve; dpi, days postinfection; dpc, day postcontact; REU, relative equivalent unit.

In study I (Figure 3, panel A), we directly infected 6 animals and then placed them in direct contact with 6 uninfected animals of the same species 1 day later. Clinical signs from H1N2r infection were unapparent or mild (1–2 days) in pigs and ferrets, whether directly infected or contact animals, and there was no major change in body temperature (<1°C above baseline). We observed clinical signs in 3 of 12 pigs: 2 pigs had mild rhinitis at 1 dpi, and 1 pig had mild respiratory signs (sneezing) at 5 dpi. Clinical signs in ferrets were limited to transient rhinitis in some animals and could have been caused by infection or other factors, such as sampling.

### Shedding and Transmission Profile of H1N2r Virus

We monitored virus RNA shedding daily in nasal swab specimens (pigs) or washes (ferrets) and quantified results by using real-time qRT-PCR. Nasal shedding started in directly infected animals at <1 dpi and in uninfected contacts at 1–2 days after contact (Figure 3, panels B, C). These results from study I confirmed transmission between infected and uninfected animals of the same species. Peak amounts of virus RNA were shed by directly infected animals at 2–5 dpi. In comparison with directly infected or contact pigs (Figure 3, panel B), directly infected or contact ferrets (Figure 3, panel C) shed virus for a longer duration and also shed a larger total quantity of virus. Significantly greater (p<0.0001) quantities of virus RNA were shed by directly infected ferrets in comparison with all other groups, and also by contact ferrets in comparison with contact pigs, as shown by area under the curve analysis (Figure 3, panel D). A greater variation in shedding occurred between individual ferrets than between pigs, particularly among contact animals.

In study II (Figure 3, panel A), directly infected pigs or ferrets (representing a human model) were cohoused with animals of the other species to assess interspecies transmission of the H1N2r virus and zoonotic potential. Direct infection of pigs (Figure 3, panel E) or ferrets (Figure 3, panel F) resulted in similar infection dynamics as for intraspecies study I. When infected ferrets were cohoused with uninfected pigs (Figure 3, panel E), the pigs became infected after a considerable 8–10-day lag, and virus shedding profiles differed between recipient pigs. These infection kinetics might indicate that some pigs became infected from their penmates, demonstrating possible onward transmission of virus and potential for the H1N2r virus to disseminate in a susceptible population. Ferrets were readily infected within 4 days when cohoused with infected pigs (Figure 3, panel F), and the infection profile was similar to that observed for intraspecies transmission, suggesting that all ferrets were infected synchronously by the infected pigs. Nasal shedding profiles observed for animals infected directly with H1N2r were comparable with profiles for human pH1N1 influenza viruses A/England/195/2009 and A/California/07/2009 (*36**,**37**,**39**,**40*).

### Serologic Analysis

**Figure 4:**
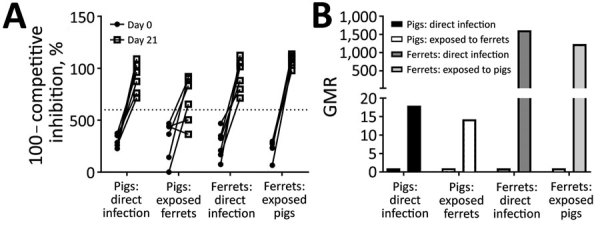
Serum antibody levels monitored in groups of pigs and ferrets in interspecies transmission study II to assess seroconversion after infection. A) Influenza A virus nucleoprotein competitive ELISA, showing inverse of competitive inhibition, %. Dotted line indicates lower limit for the positive threshold. B) GMR of hemagglutination–inhibition titer to the A/swine/England/1382/2010 challenge strain. GMR, geometric mean ratio.

Serologic analysis by competitive ELISA for NP, which is present within virions, demonstrated that, in interspecies transmission study II, infected and contact ferrets seroconverted by 21 dpi/days after contact (Figure 4, panel A). Most infected pigs also seroconverted and showed lower final antibody titers, although 2 of 6 pigs cohoused with infected ferrets did not seroconvert within the study period. HI titers, which measure exposure to the viral envelope HA protein, were positive against the H1N2r challenge strain, indicating seroconversion of all infected or contact animals (Figure 4, panel B). Both assays showed that the antibody response was stronger in ferrets than pigs, perhaps corresponding to the higher virus load and more prolonged infection in ferrets.

## Discussion

Our study showed that pigs and ferrets were susceptible to infection with the H1N2r virus and showed clinical signs and virologic parameters indicating mild disease. Longitudinal postmortem analysis indicated that this virus could be more adapted to a swine host because infection disseminated more rapidly throughout the respiratory tract, and distribution of infection in the lung on all postmortem days was more evident in pigs than in ferrets. However, in contrast to pigs, ferrets shed a larger total quantity of virus for an extended duration, and seroconversion occurred in all infected and contact ferrets by 21 dpi. However, interpretation of data is limited by the different intranasal infection protocols and nasal sampling techniques for the 2 species. All animals had increased specific antibody titers postinfection. However, in comparison with ferrets, titers in pigs were lower and, in some contact pigs, were below the positive threshold. These findings are comparable with outcomes reported for pH1N1 virus infection of pigs and ferrets (*36*,*37**,**39*,*40*) and a comparison of swine H1 viruses, including H1N2v strains, when infecting ferrets (*41*). A North American reassortant H1N1 swine IAV incorporating pH1N1-origin polymerase acidic, NP, and M genes also reportedly showed similar replication kinetics to a swine IAV pH1N1 isolate when infecting ferrets (*14*). We conclude that there are no apparent phenotypic changes associated with the novel combination of genes in the H1N2r reassortant virus.

Intraspecies transmission by direct contact was demonstrated in our study in pigs and ferrets. Previous studies have focused on ferret-to-ferret transmission of reassortant viruses as a means of assessing risk because the ferret is an accepted small animal model for human influenza, and this animal has comparable anatomic properties, such as respiratory tract distribution of virus receptors, clinical manifestations, and transmission patterns (*25*). The reassortant H1N2 virus isolated in Denmark, incorporates all pH1N1 genes except the NA segment (*10*). However, ferret-to-ferret transmission consistently occurred only by direct contact and not by the airborne respiratory droplet route. In contrast, an H1N2 strain from South Korea that had the pH1N1 internal gene cassette was found to infect directly inoculated and contact ferrets and also be transmitted at a lower efficiency, to ferrets indirectly exposed to airborne respiratory droplets (*42*). In a similar fashion, an H1N2 reassortant strain from Chile containing the pH1N1 internal gene cassette was also transmitted between ferrets by direct and indirect routes (*19*).

Swine are known to support replication of viruses with a wide range of HA activation pH values, whereas ferrets support replication of a narrower pH activation range for HA (*40*). In addition, it has been suggested that successful transmission of influenza A viruses requires a balance between the HA and NA activities (*43*). Therefore, the HA and NA combination of the H1N2r isolate that we studied is compatible with replication and transmission in both mammalian hosts.

In our study, we modeled the zoonotic and reverse-zoonotic infection potential of the H1N2r virus by investigating virus transmission from infected pigs or infected ferrets to cohoused animals of the other species. Transmission by the indirect respiratory droplet route occurred, whether pigs or ferrets were the infection source. Transmission of virus from pigs occurred more rapidly in comparison with ferrets. This finding could have occurred as a consequence of the room layout, the different degree of aerosolization of virus shed from ferrets or pigs, or the anatomic and physiologic differences in the pig and ferret nasal tract. Once animals became infected, whether directly or indirectly, the shedding profile was consistent within the same species. These findings indicate that the threat associated with pH1N1 reassortant viruses from swine or human origin is no greater from either donor species, although zoonotic infection is clearly plausible and might be augmented by the presence of the pH1N1 internal gene cassette.

In conclusion, our study demonstrates that a swine H1N2r virus can be readily transmitted between mammalian species. Although this virus does not display enhanced virulence compared with other swine IAV or human pH1N1 viruses (*36**,**39*), it nevertheless shows high interspecies and intraspecies transmissibility. This virus strain represents a newly emergent reassortant virus that could enhance the genetic diversity of circulating strains and contribute to influenza A virus genotypic change at the human–animal interface, thereby increasing the potential for generating new viruses with altered disease phenotype or fitness for new host species.

Appendix Additional information on interspecies transmission of swine influenza A virus containing genes from influenza A(H1N1)pdm09 and swine influenza A(H1N2) viruses.

## References

[1] Brown IH. History and epidemiology of Swine influenza in Europe. Curr Top Microbiol Immunol. 2013;370:133–46. 10.1007/82_2011_194 22234411

[2] Smith GJ, Vijaykrishna D, Bahl J, Lycett SJ, Worobey M, Pybus OG, et al. Origins and evolutionary genomics of the 2009 swine-origin H1N1 influenza A epidemic. Nature. 2009;459:1122–5. 10.1038/nature08182 19516283

[3] Anderson TK, Macken CA, Lewis NS, Scheuermann RH, Van Reeth K, Brown IH, et al. A phylogeny-based global nomenclature system and automated annotation tool for H1 hemagglutinin genes from swine influenza A viruses. MSphere. 2016;1:e00275–16. 10.1128/mSphere.00275-16 27981236 PMC5156671

[4] Bhatt S, Lam TT, Lycett SJ, Leigh Brown AJ, Bowden TA, Holmes EC, et al.; Combating Swine Influenza Consortium. The evolutionary dynamics of influenza A virus adaptation to mammalian hosts. Philos Trans R Soc Lond B Biol Sci. 2013;368:20120382. 10.1098/rstb.2012.0382 23382435 PMC3678336

[5] Lewis NS, Russell CA, Langat P, Anderson TK, Berger K, Bielejec F, et al.; ESNIP3 consortium. The global antigenic diversity of swine influenza A viruses. eLife. 2016;5:e12217. 10.7554/eLife.12217 27113719 PMC4846380

[6] Watson SJ, Langat P, Reid SM, Lam TT, Cotten M, Kelly M, et al.; ESNIP3 Consortium. ESNIP3 Consortium. Molecular epidemiology and evolution of influenza viruses circulating within European swine between 2009 and 2013. J Virol. 2015;89:9920–31. 10.1128/JVI.00840-15 26202246 PMC4577897

[7] Henritzi D, Hoffmann B, Wacheck S, Pesch S, Herrler G, Beer M, et al. A newly developed tetraplex real-time RT-PCR for simultaneous screening of influenza virus types A, B, C and D. Influenza Other Respir Viruses. 2018. 10.1111/irv.12613 PMC6304318 30264926

[8] Howard WA, Essen SC, Strugnell BW, Russell C, Barass L, Reid SM, et al. Reassortant Pandemic (H1N1) 2009 virus in pigs, United Kingdom. Emerg Infect Dis. 2011;17:1049–52. 10.3201/eid/1706.101886 21749767 PMC3358214

[9] Moreno A, Di Trani L, Faccini S, Vaccari G, Nigrelli D, Boniotti MB, et al. Novel H1N2 swine influenza reassortant strain in pigs derived from the pandemic H1N1/2009 virus. Vet Microbiol. 2011;149:472–7. 10.1016/j.vetmic.2010.12.011 21208754

[10] Fobian K, Fabrizio TP, Yoon SW, Hansen MS, Webby RJ, Larsen LE. New reassortant and enzootic European swine influenza viruses transmit efficiently through direct contact in the ferret model. J Gen Virol. 2015;96:1603–12. 10.1099/vir.0.000094 25701826 PMC4635450

[11] Starick E, Fereidouni SR, Lange E, Grund C, Vahlenkamp T, Beer M, et al. Analysis of influenza A viruses of subtype H1 from wild birds, turkeys and pigs in Germany reveals interspecies transmission events. Influenza Other Respir Viruses. 2011;5:276–84. 10.1111/j.1750-2659.2011.00201.x 21651738 PMC4634544

[12] Krog JS, Hjulsager CK, Larsen MA, Larsen LE. Triple-reassortant influenza A virus with H3 of human seasonal origin, NA of swine origin, and internal A(H1N1) pandemic 2009 genes is established in Danish pigs. Influenza Other Respir Viruses. 2017;11:298–303. 10.1111/irv.12451 28245096 PMC5410721

[13] Vijaykrishna D, Poon LL, Zhu HC, Ma SK, Li OT, Cheung CL, et al. Reassortment of pandemic H1N1/2009 influenza A virus in swine. Science. 2010;328:1529. 10.1126/science.1189132 20558710 PMC3569847

[14] Ducatez MF, Hause B, Stigger-Rosser E, Darnell D, Corzo C, Juleen K, et al. Multiple reassortment between pandemic (H1N1) 2009 and endemic influenza viruses in pigs, United States. Emerg Infect Dis. 2011;17:1624–9. 10.3201/1709.110338 21892996 PMC3322089

[15] Nelson MI, Schaefer R, Gava D, Cantão ME, Ciacci-Zanella JR, Influenza A. Influenza A viruses of human origin in swine, Brazil. Emerg Infect Dis. 2015;21:1339–47. 10.3201/eid2108.141891 26196759 PMC4517702

[16] Biondo N, Schaefer R, Gava D, Cantão ME, Silveira S, Mores MA, et al. Genomic analysis of influenza A virus from captive wild boars in Brazil reveals a human-like H1N2 influenza virus. Vet Microbiol. 2014;168:34–40. 10.1016/j.vetmic.2013.10.010 24238665

[17] Schaefer R, Rech RR, Gava D, Cantão ME, da Silva MC, Silveira S, et al. A human-like H1N2 influenza virus detected during an outbreak of acute respiratory disease in swine in Brazil. Arch Virol. 2015;160:29–38. 10.1007/s00705-014-2223-z 25209152

[18] Resende PC, Born PS, Matos AR, Motta FC, Caetano BC, Debur MD, et al. Whole-genome characterization of a novel human influenza A(H1N2) virus variant, Brazil. Emerg Infect Dis. 2017;23:152–4. 10.3201/eid2301.161122 27983507 PMC5176240

[19] Bravo-Vasquez N, Karlsson EA, Jimenez-Bluhm P, Meliopoulos V, Kaplan B, Marvin S, et al. Swine influenza virus (H1N2) characterization and transmission in ferrets, Chile. Emerg Infect Dis. 2017;23:241–51. 10.3201/eid2302.161374 28098524 PMC5324791

[20] Komadina N, McVernon J, Hall R, Leder K. A historical perspective of influenza A(H1N2) virus. Emerg Infect Dis. 2014;20:6–12. 10.3201/eid2001.121848 24377419 PMC3884707

[21] Pascua PN, Lim GJ, Kwon HI, Kim YI, Kim EH, Park SJ, et al. Complete genome sequences of novel reassortant H1N2 swine influenza viruses isolated from pigs in the Republic of Korea. Genome Announc. 2013;1:e00552–13. 10.1128/genomeA.00552-13 23929468 PMC3738884

[22] Peng X, Wu H, Xu L, Peng X, Cheng L, Jin C, et al. Molecular characterization of a novel reassortant H1N2 influenza virus containing genes from the 2009 pandemic human H1N1 virus in swine from eastern China. Virus Genes. 2016;52:405–10. 10.1007/s11262-016-1303-4 26980674

[23] Williamson SM, Tucker AW, McCrone IS, Bidewell CA, Brons N, Habernoll H, et al.; COSI. Descriptive clinical and epidemiological characteristics of influenza A H1N1 2009 virus infections in pigs in England. Vet Rec. 2012;171:271. 10.1136/vr.100673 22865115

[24] Fragaszy E, Ishola DA, Brown IH, Enstone J, Nguyen-Van-Tam JS, Simons R, et al.; Flu Watch Group; Combating Swine Influenza (COSI) Consortium. Increased risk of A(H1N1)pdm09 influenza infection in UK pig industry workers compared to a general population cohort. Influenza Other Respir Viruses. 2016;10:291–300. 10.1111/irv.12364 26611769 PMC4910179

[25] Belser JA, Katz JM, Tumpey TM. The ferret as a model organism to study influenza A virus infection. Dis Model Mech. 2011;4:575–9. 10.1242/dmm.007823 21810904 PMC3180220

[26] Trock SC, Burke SA, Cox NJ. Development of framework for assessing influenza virus pandemic risk. Emerg Infect Dis. 2015;21:1372–8. 10.3201/eid2108.141086 26196098 PMC4517742

[27] Brauer R, Chen P. Influenza virus propagation in embryonated chicken eggs. J Vis Exp. 2015;•••. 10.3791/52421 25867050 PMC4401370

[28] Hemmink JD, Morgan SB, Aramouni M, Everett H, Salguero FJ, Canini L, et al. Distinct immune responses and virus shedding in pigs following aerosol, intra-nasal and contact infection with pandemic swine influenza A virus, A(H1N1)09. Vet Res (Faisalabad). 2016;47:103. 10.1186/s13567-016-0390-5 27765064 PMC5073419

[29] Baillie GJ, Galiano M, Agapow PM, Myers R, Chiam R, Gall A, et al. Evolutionary dynamics of local pandemic H1N1/2009 influenza virus lineages revealed by whole-genome analysis. J Virol. 2012;86:11–8. 10.1128/JVI.05347-11 22013031 PMC3255882

[30] Brown IH, Harris PA, Alexander DJ. Serological studies of influenza viruses in pigs in Great Britain 1991-2. Epidemiol Infect. 1995;114:511–20. 10.1017/S0950268800052225 7781739 PMC2271297

[31] Brown IH, Harris PA, McCauley JW, Alexander DJ. Multiple genetic reassortment of avian and human influenza A viruses in European pigs, resulting in the emergence of an H1N2 virus of novel genotype. J Gen Virol. 1998;79:2947–55. 10.1099/0022-1317-79-12-2947 9880008

[32] van den Brand JM, Stittelaar KJ, van Amerongen G, Reperant L, de Waal L, Osterhaus AD, et al. Comparison of temporal and spatial dynamics of seasonal H3N2, pandemic H1N1 and highly pathogenic avian influenza H5N1 virus infections in ferrets. PLoS One. 2012;7:e42343. 10.1371/journal.pone.0042343 22905124 PMC3414522

[33] Slomka MJ, Densham AL, Coward VJ, Essen S, Brookes SM, Irvine RM, et al. Real time reverse transcription (RRT)-polymerase chain reaction (PCR) methods for detection of pandemic (H1N1) 2009 influenza virus and European swine influenza A virus infections in pigs. Influenza Other Respir Viruses. 2010;4:277–93. 10.1111/j.1750-2659.2010.00149.x 20716157 PMC4634650

[34] Löndt BZ, Nunez A, Banks J, Nili H, Johnson LK, Alexander DJ. Pathogenesis of highly pathogenic avian influenza A/turkey/Turkey/1/2005 H5N1 in Pekin ducks (*Anas platyrhynchos*) infected experimentally. Avian Pathol. 2008;37:619–27. 10.1080/03079450802499126 19023759

[35] Kitikoon P, Gauger PC, Vincent AL. Hemagglutinin inhibition assay with swine sera. Methods Mol Biol. 2014;1161:295–301. 10.1007/978-1-4939-0758-8_24 24899438

[36] Brookes SM, Núñez A, Choudhury B, Matrosovich M, Essen SC, Clifford D, et al. Replication, pathogenesis and transmission of pandemic (H1N1) 2009 virus in non-immune pigs. PLoS One. 2010;5:e9068. 10.1371/journal.pone.0009068 20140096 PMC2816721

[37] Vidaña B, Martínez J, Martínez-Orellana P, García Migura L, Montoya M, Martorell J, et al. Heterogeneous pathological outcomes after experimental pH1N1 influenza infection in ferrets correlate with viral replication and host immune responses in the lung. Vet Res (Faisalabad). 2014;45:85. 10.1186/s13567-014-0085-8 25163545 PMC4161856

[38] Martínez-Orellana P, Martorell J, Vidaña B, Majó N, Martínez J, Falcón A, et al. Clinical response to pandemic H1N1 influenza virus from a fatal and mild case in ferrets. Virol J. 2015;12:48. 10.1186/s12985-015-0272-x 25888921 PMC4380011

[39] Janke BH. Influenza A virus infections in swine: pathogenesis and diagnosis. Vet Pathol. 2014;51:410–26. 10.1177/0300985813513043 24363301

[40] Russier M, Yang G, Marinova-Petkova A, Vogel P, Kaplan BS, Webby RJ, et al. H1N1 influenza viruses varying widely in hemagglutinin stability transmit efficiently from swine to swine and to ferrets. PLoS Pathog. 2017;13:e1006276. 10.1371/journal.ppat.1006276 28282440 PMC5362248

[41] Pulit-Penaloza JA, Pappas C, Belser JA, Sun X, Brock N, Zeng H, et al. Comparative in vitro and in vivo analysis of H1N1 and H1N2 variant influenza viruses isolated from humans between 2011 and 2016. J Virol. 2018;92:e01444–18. 10.1128/JVI.01444-18 30158292 PMC6206486

[42] Lee JH, Pascua PN, Decano AG, Kim SM, Park S-J, Kwon H-I, et al. Evaluation of the zoonotic potential of a novel reassortant H1N2 swine influenza virus with gene constellation derived from multiple viral sources. Infect Genet Evol. 2015;34:378–93. 10.1016/j.meegid.2015.06.005 26051886

[43] Yen H-L, Liang C-H, Wu C-Y, Forrest HL, Ferguson A, Choy K-T, et al. Hemagglutinin-neuraminidase balance confers respiratory-droplet transmissibility of the pandemic H1N1 influenza virus in ferrets. Proc Natl Acad Sci U S A. 2011;108:14264–9. 10.1073/pnas.1111000108 21825167 PMC3161546

