# Supplementary material for: Interspecies Transmission of Reassortant Swine Influenza A Virus Containing Genes from Swine Influenza A(H1N1)pdm09 and A(H1N2) Viruses
Source: Emerg Infect Dis. 2020 Feb;26(2):273–81. doi: 10.3201/eid2602.190486 (PMC6986826; doi:10.3201/eid2602.190486)
Supplement: Appendix — Additional information on interspecies transmission of swine influenza A virus containing genes from influenza A(H1N1)pdm09 and swine influenza A(H1N2) viruses. [file 19-0486-Techapp-s1.pdf]

Article DOI: https://doi.org/10.3201/eid2601.190486

Interspecies Transmission of Reassortant Swine Influenza A Virus Containing Genes from Swine Influenza A(H1N1)pdm09 and A(H1N2) Viruses

Appendix

Organ Culture Preparation

Ferret tracheal cultures were prepared as follows. petri dishes were prepared with plugs of 1% (wt/vol) low-gelling temperature agarose (Sigma, https://www.sigmaaldrich.com) in water. Plugs were immersed in Dulbecco modified Eagle medium (DMEM) medium containing 2 mol/L L-glutamine and antimicrobial drugs (200 U/mL penicillin, 2,000 μg/mL streptomycin, and 2.5 μg/mL amphotericin B (all from GIBCO, https://www.thermofisher.com), then incubated at 37°C in an atmosphere of 5% CO_2. Tracheal tissue was immersed in prewarmed, supplemented DMEM and incubated at 37°C in an atmosphere of 5% CO_2 for 20 min. The medium was replaced ≥3 times, and the tissue was dissected and placed onto sterile filter paper overlaying an agarose plug, with the filter paper forming a wick into the medium.

To culture lung tissue, a lung lobe was filled through the bronchi with phosphate-buffered saline supplemented with antimicrobial drugs and maintained at 37°C. Low-gelling temperature agarose (1% and 4% [wt/vol] in phosphate-buffered saline) was equilibrated to 38°C. The lung lobe was filled with 1% agarose and set with 4% agarose inside a syringe. Once solidified, the top of the syringe was removed, the lung extruded, and 3–4-mm sections were cut and incubated 18 h in supplemented DMEM at 37°C in an atmosphere of 5% CO_2. Sections were moved to a 6-well plate, and the outer 4% agarose layer was removed.

Positive Strand-Specific PCR

Positive-sense RNA encoding the virus matrix protein gene was quantified to assess levels of virus replication. Reverse transcription was conducted by using the M-MLV Reverse Transcription Kit (Promega, https://www.promega.com) and reverse primer NcRev1 (5′-AGTAGAAACAAGGTAGTTTT-3′) at a final concentration of 2.5 μmol/L. Real-time PCR was

conducted by using the Quantifast Kit (QIAGEN, https://www.qiagen.com) according to the manufacturer's specifications using the forward and reverse primers M808F (5′-GCAGATGCAGCGATTCAAG-3′) and swM1003R (5′-CACTCTGCTGTTCCTGTTGATAT-3′) at final concentrations of 0.04 μmol/L and the probe M945RPro (FAM-5′-AGGCCCTCTTTTCAAACCGTATT-3′-TAMRA at a final concentration of 0.02 μmol/L. Cycling parameters for an MxPro3000 Instrument (Agilent, https://www.agilent.com) were 1 cycle of 95°C for 3 min and 40 cycles at 94°C for 10 s, 55°C for 30 s, and 72°C for 30 s. Data readings were collected during annealing and analyzed by using MxPro software (Agilent).

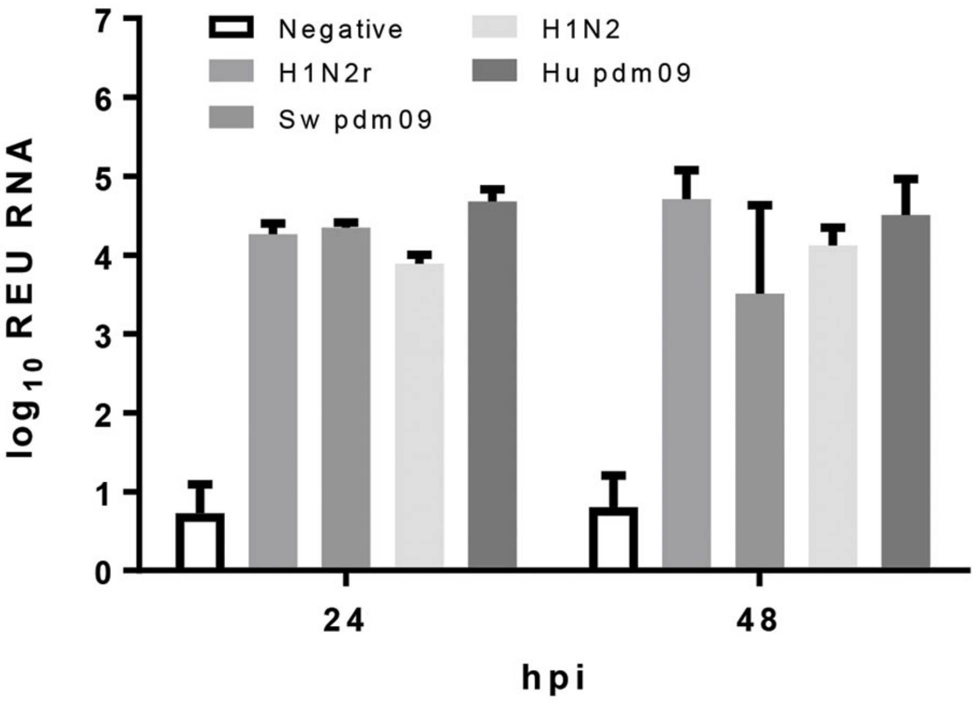

Appendix Figure. Infection and replication of swine H1N2r virus in ferret lung organ cultures. Quantity of influenza A virus RNA in ferret lung ex vivo organ cultures at 24 h and 48 h postinoculation with swine viruses H1N2r (A/swine/England/1382/2010), Sw pdm09 (A/swine/England/1353/2009), H1N2 (A/swine/England/997/2009), and human isolate Hu pdm09 (A/England/195/2009). Results are log_{10} REU in combined supernatants and tissue lysates for each sample. Error bars indicate SEM for triplicate cultures. hpi, hours postinoculation; Hu, human; H1N2r, reassortant swine influenza A virus; pdm, pandemic; REU, relative equivalent unit; Sw, swine.
